# Rapid transitions in the standard of care for chronic lymphocytic leukemia (CLL)

**DOI:** 10.18632/oncotarget.26826

**Published:** 2019-04-02

**Authors:** Erlene K. Seymour

**Affiliations:** Department of Oncology, Karmanos Cancer Institute, Wayne State University, Detroit, MI, USA

**Keywords:** chronic lymphocytic leukemia, treatment patterns, cost of care, toxicities, ibrutinib

Major changes in CLL therapy occurred in the last two decades, including the addition of rituximab (anti-CD20 monoclonal antibody) to fludarabine, cyclophosphamide or bendamustine, such that "chemoimmunotherapy" (CIT) became the standard of care. Diagnostic testing identified patients in whom CIT was less effective, such as those with deletion 17p/p53 mutations. In contrast, patients with mutated IgVH have prolonged progression free survival (PFS) lasting more than 10 years when treated with fludarabine, cyclophosphamide, and rituximab (FCR). A study comparing rituximab and bendamustine (BR) *versus* FCR in untreated patients demonstrated prolonged PFS with FCR among patients < 65 years, but no difference in PFS among older patients (reviewed in [1]). We recently evaluated CLL treatment trends using the SEER Patterns of Care dataset from 2008-2016 [2]. In this period, CIT remained the standard frontline therapy, but within 5 years, BR had increased its use at the expense of FCR, particularly among older patients and those with comorbidities. We observed a significant increase in use of FISH testing and IgVH mutation analyses over time, particularly in teaching hospitals compared to non-teaching hospitals.

CLL therapy recently evolved again, with ibrutinib (Bruton tyrosine kinase inhibitor), idelalisib (PI3 kinase inhibitor), and venetoclax (BCL-2 inhibitor) demonstrating improved clinical outcomes among patients with deletion 17p, and durable responses for most CLL patients. These were studied among patients with relapsed/refractory CLL, and subsequently evaluated as initial treatment in older and younger patients. As our SEER manuscript was in press, I watched the plenary and late-breaking abstract presentations of randomized prospective trials comparing ibrutinib *versus* CIT during the American Society of Hematology Annual Meeting in December 2018. In these trials, ibrutinib demonstrated a superior progression free survival compared to BR among older CLL patients [3] and as well as a superior overall survival and progression free survival relative to FCR among younger patients [4].

This lends itself to easy predictions about future patterns of care. The use of ibrutinib will increase in the frontline treatment of most patients. It already replaced BR in some practices because of its toxicity profile and convenient oral formulation. The demonstration of superior PFS reinforces this trend. There may be a tendency for some clinicians to consider testing unnecessary since ibrutinib is indicated for most patients. FISH testing should be done to identify del17p patients, since these patients have shorter PFS and OS with ibrutinib monotherapy than patients without del17p (median 26 and 57 months respectively [5]), and venetoclax and PI3 kinases could also be considered. The clinical situation which remains in limbo is FCR *versus* ibrutinib among young patients with mutated IgVH. For these patients, there was no difference in PFS, and treatment decisions should be discussed with regard to their toxicity and convenience of therapy. For this reason, IgVH mutation testing should be performed, particularly for younger patients who are deciding whether to take chronic ibrutinib therapy or have a "one and done" approach with a course of FCR.

The toxicity and cost differences between CIT and ibrutinib are notable. FCR carries a risk of opportunistic infections, hemolytic anemia, and secondary malignancies. However, the chronic Grade 1-2 toxicities with ibrutinib, including atrial fibrillation, arthralgias, and infections, can be life-disrupting [6]. Few patients on ibrutinib achieve complete remission, and mechanisms of resistance have already been identified. The annual drug price of ibrutinib 420 mg daily is ~$175,000 in the U.S., with an estimated cost-sharing (for a Medicare patient) of $8000-11,000/year [7] for a treatment continued indefinitely. In a survey study, when given the choice of two hypothetical CLL drugs, one which cost $400 more per month but with a longer PFS compared to another drug, most patients still chose the lower costing drug, demonstrating cost influence on treatment decision-making [8].

Since chronic toxicities and cost continue to be a burden, newer trials have been designed to test chemotherapy-free combination therapies of limited duration. Combination therapies are being used to achieve cytoreduction to nondetectable minimal residual disease which predicts longer PFS [9, 10] and which may translate to longer time to subsequent treatment with longer overall survival. An Alliance trial comparing the "one and done" approach of combination obinutuzumab + venetoclax + ibrutinib over 15 months [10], compared to chronic therapy with ibrutinib + obinutuzumab has recently been initiated in the US, and will be of great interest to compare toxicities and clinical outcomes.

The full BTK receptor occupancy of ibrutinib is demonstrated at 2.5 mg/kg/d, however standard dosing for CLL is 3-fold higher [11]. In a recent study, the efficacy of starting with one month of 420 mg daily dosing, then decreasing to 140 mg daily, demonstrated no loss of biological efficacy in a small cohort of patients [12]. As these results were presented, flat pricing was introduced for a new formulation of single pill 140 mg, 280 mg, 420 mg, and 560 mg tablets. After an editorial by Ratain and colleagues [13], and community concerns in delaying dose adjusting and increasing cost for patients, the original 140 mg capsules returned to formularies. This was one of several examples of how dosing was based on smaller studies with an emphasis on maximum tolerated dose (MTD), without initially studying equivalence at lower doses. A recommendation to proceed with a larger prospective trial comparing standard to lower doses was strongly encouraged.

These are examples of how trial design can be focused on reducing chronic toxicity and cost, while maintaining and/or improving clinical outcomes. For novel oral therapies, essential interventions in reducing patient cost will be negotiating drug prices and optimizing insurance design so cost-sharing is minimized. However, these policy and pricing decisions made by a few individuals appear out-of-reach to oncologists. But as we demonstrated within CLL, we are all persuasive advocates for our patients in this matter. Attentive dosing and trial design, as well as discussing cost with our patients, are modest but important steps toward making these impossible goals possible. Ibrutinib should not only be an advancement of standard of care in CLL, but hopefully a tipping point on how we transition from chemotherapy to novel agents, as we enter a renaissance of chemotherapy-free options for multiple cancers.
